
JOURNAL INFORMATION
==============================
NLM Title Abbreviation: Eur Psychiatry
Journal ID: Eur Psychiatry
NLM Journal ID: 9111820
Journal ID: EPA

European Psychiatry

ISSN: 0924-9338
EISSN: 1778-3585
Publisher: Cambridge University Press

ARTICLE INFORMATION
==============================
PMCID: PMC9412710
DOI: 10.1192/j.eurpsy.2020.4
Article ID: S0924933820000048
Article version: 1
Subjects: Abstracts

Debate

Publication date: 2020 Jul
Electronic publication date: 2020 Sep 4
Volume: 63
Issue: Suppl 1
Issue title: Abstracts of the 28th European Congress of Psychiatry
First page: S1
Last page: S2
Copyright: © The Author(s) 2020
Copyright year: 2020
Copyright holder: The Author(s)
License: This is an Open Access article, distributed under the terms of the Creative Commons Attribution licence (http://creativecommons.org/licenses/by/4.0/), which permits unrestricted re-use, distribution, and reproduction in any medium, provided the original work is properly cited.
License URL: https://creativecommons.org/licenses/by/4.0/

Page count: 2

==============================

Article ID: D001
Article ID: Pro

Antidepressants in bipolar depression: should we use them?

Pacchiarotti I.
Bipolar and Depressive Disorders Unit, Hospital Clínic de Barcelona, Psychiatry , Barcelona , Spain

Article ID: D003
Article ID: Pro

Do EE need antipsychotics for the long-term treatment of Schizophrenia?

Emsley R.
Stellenbosch University , Psychiatry, Cape Town , South Africa

Article ID: D005
Article ID: Pro

Is Psychotherapy The First Line of Treatment for Borderline Personality Disorder?

Claes L.
University of Leuven , Psychology and Educational Sciences, Leuven , Belgium

